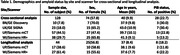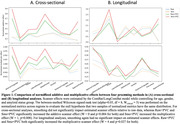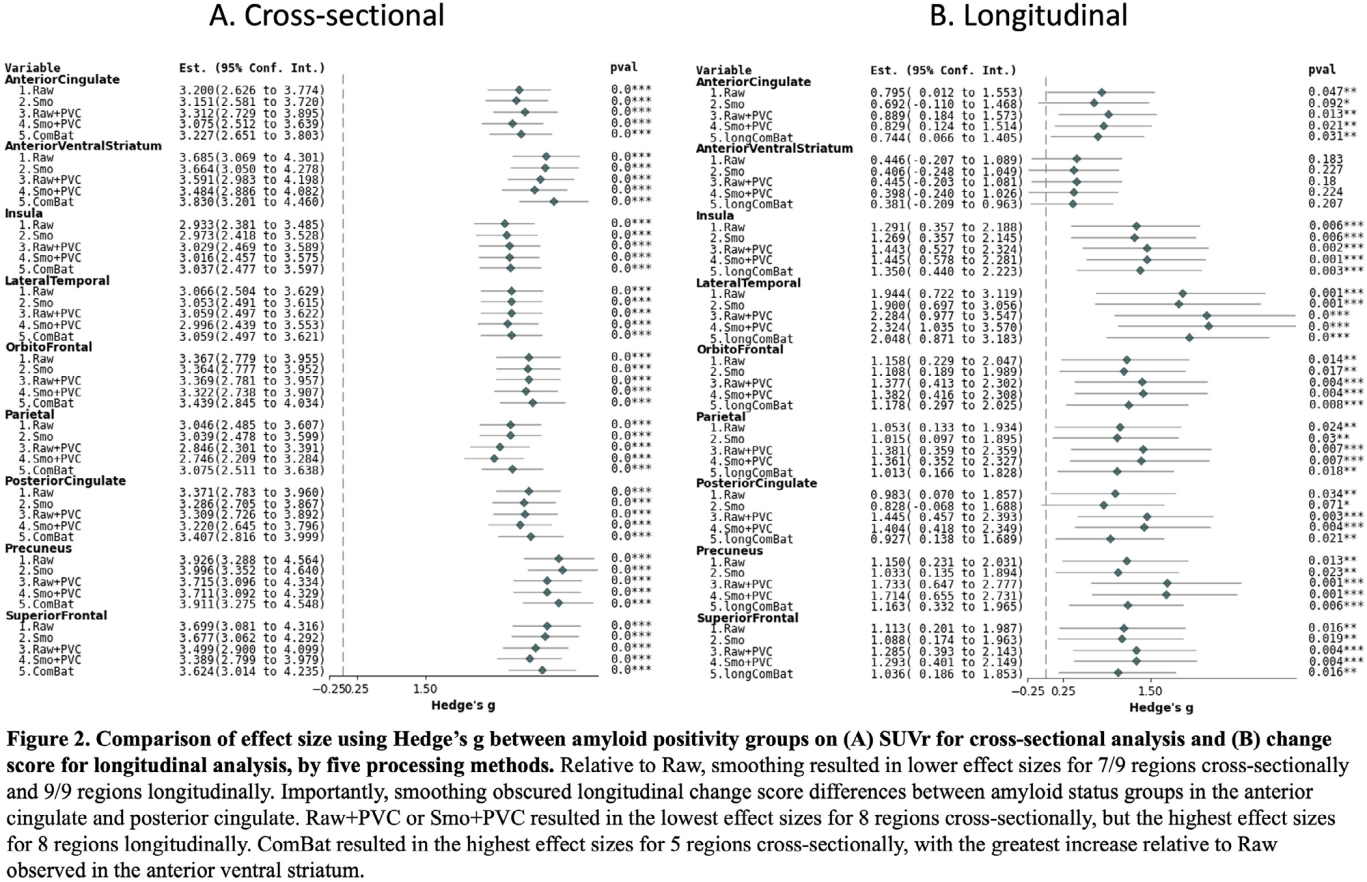# Evaluation of image processing methods on amyloid PET scanner effects for multisite cross‐sectional and longitudinal studies of Down syndrome

**DOI:** 10.1002/alz.092929

**Published:** 2025-01-09

**Authors:** Weiquan Luo, Charles M Laymon, Dana Tudorascu, Ann D Cohen, Beau Ances, Shahid Zaman, Bradley T. Christian, William E Klunk, Benjamin L Handen, Davneet S Minhas

**Affiliations:** ^1^ University of Pittsburgh, Pittsburgh, PA USA; ^2^ Washington University in St. Louis School of Medicine, St. Louis, MO USA; ^3^ University of Cambridge, Cambridge UK; ^4^ University of Wisconsin‐Madison, Madison, WI USA

## Abstract

**Background:**

Gaussian smoothing to a common image resolution is frequently employed to harmonize Aβ PET in multisite studies. However, spatial smoothing of PET can increase spill‐over contamination between neighboring regions. Geometric transfer matrix partial volume correction (PVC) has been applied, in turn, to correct for such contamination. Despite being common practices, the impact of smoothing and PVC on scanner effects remains unclear for cross‐sectional and longitudinal outcomes.

We applied four putative image processing and statistical harmonization methods to unharmonized, multisite ^11^C‐PiB PET data from the ABC‐DS (^11^C‐PiB only) Down syndrome dataset to evaluate their impact on amyloid status group separation in cross‐sectional and longitudinal analyses.

**Method:**

^11^C‐PiB images (Raw) from 4 different PET scanners (Table 1) were included in the analyses. Putative image‐processing harmonization methods evaluated were: (1) smoothing of PET images to an effective resolution of 8mm (Smo); (2) application of PVC to raw PET (Raw+PVC); (3) application of PVC to smoothed PET (Smo+PVC); and (4) application of ComBat/LongComBat to cross‐sectional/longitudinal Raw regional values.

SUVR outcomes were extracted for all methods from 9 Aβ‐relevant regions. Mean normalized additive and multiplicative scanner effects were assessed for image‐processing method outcomes using ComBat/LongComBat frameworks. Hedge’s g effect sizes were evaluated for all image‐processing and ComBat/LongComBat methods between Aβ status groups cross‐sectionally on regional SUVR outcomes and longitudinally on change score outcomes between baseline and follow‐up SUVRs.

**Result:**

Estimated scanner effects across image‐processing methods are presented in Figure 1. Smoothing did not decrease scanner effects for any analyses, while Raw+PVC and Smo+PVC tended to increase scanner effects.

Effect sizes across methods, including ComBat and LongComBat, are presented in Figure 2. Cross‐sectionally, ComBat outperformed all other methods in 5/9 regions, with the greatest increase in effect size observed in the anterior ventral striatum. Longitudinally, Raw+PVC or Smo+PVC resulted in the greatest effect sizes in 8/9 regions. Importantly, smoothing reduced longitudinal effect sizes in 9/9 regions relative to Raw and obscured group differences in the anterior and posterior cingulates.

**Conclusion:**

Gaussian smoothing may not address ^11^C‐PiB PET scanner effects estimated using ComBat/LongComBat frameworks. Spatial smoothing, in fact, may confound the ability to differentiate longitudinal change between groups.